# The impact of accelerating population aging on service industry development: Evidence from China

**DOI:** 10.1371/journal.pone.0296623

**Published:** 2024-06-06

**Authors:** Mingzhi Zhang, Chao Chen, Xiangyu Zhou, Xinpei Wang, Bowen Wang, Fuying Huan, Jianxu Liu

**Affiliations:** 1 School of Economics, Shandong University of Finance and Economics, Jinan, China; 2 Institute of Population and Economic Development, Shandong University of Finance and Economics, Jinan, China; Qufu Normal University, CHINA

## Abstract

The demographic structure is an important factor influencing the development of the services industry. As the country with the world’s most serious aging problem, China’s service industry structure is likely to undergo profound changes in response to the rapid demographic transition. Therefore, this paper examines the effect of population aging on the development of the service industry in the context of China’s accelerating population aging. The study found that: (1) Population aging has a significant "inverted U" effect on the development of the services industry. (2) The impact of population aging on the development of the service industry has obvious regional and industry heterogeneity. The study of regional heterogeneity found that population aging in economically developed regions has a more obvious effect on the development of the service industry than in economically less developed regions. Industry heterogeneity studies found that population aging has an obvious promotional effect on the development of medical and other rigid demand industries, while the effect on other non-rigid demand industries is not significant. (3) The threshold effect test found that when the degree of population aging exceeds the threshold, the stimulating effect of population aging on the development of the services industry is no longer significant. The research in this paper provides useful insights into the likely response to changes in the industrial structure of the services industry, and offers some implications for countries with similar demographic profiles to China.

## Section 1: Introduction

Among developing countries, China has one of the most serious aging problems. Moreover, China’s population aging is characterised by both a large aging population and a rapid aging rate [1]. According to data from the seventh population census released by the National Bureau of Statistics of China, as of 1 November 2020, China’s elderly population aged 60 and above reached 264 million, accounting for 18.7% of the total population. Another 190 million people were aged 65 and above, accounting for 13.5% of the total population. China has entered a profoundly aging society by 2021. Given that the first baby boom occurred in the 1960s, this means that China will have a rapidly aging population for more than a decade after 2023, ushering in a new and even more intense period of an aging society [2, 3].

In the critical period when China’s economy is shifting from a stage of high-speed growth to a stage of high-quality development, it is particularly important for the high-quality development of China’s economy to effectively clarify the epoch-making characteristics of the development of the service industry in the new period. With the deepening of the aging of the world’s population, the research on the relationship between the aging of the population and the development of the service industry has received great attention from many scholars. However, the conclusions of the studies on the impact of population aging on the development of the service industry are not consistent. The main reason for this is that different researchers have focused on different factors affecting the development of aging services. From the perspective of the demand effect of population aging, Estrada found that population aging can increase domestic demand and thus promote the development of the service industry [4]. Maveov et al. found that as the population ages, the demand effect of an aging population on healthcare and other industries will lead to the rapid development of the service industry [5]. However, some researchers have suggested the opposite. For example, Mason et al. found that population aging and population consumption levels are negatively correlated [6]. With regard to the supply effect of aging, most researchers have found that as the degree of aging increases, the number of age-equivalent workers decreases and labor costs increase [7, 8]. Research is still divided on the impact of population aging on human capital. Some researchers have found that population aging reduces the capital stock and lowers labor productivity, which in turn limits the development of the service industry [7, 9]. Other researchers have found that population aging promotes human capital accumulation. Fougère and Merette analysed the human capital mechanism using an iteration model, and found that population aging leads to more human capital investment opportunities for future generations, promoting human capital accumulation [10]. Pekka found that with the deepening of population aging, the experience effect of aging population will be able to promote the development of the service industry by improving productivity [11]. Due to different research contents and backgrounds, it can be seen that different researchers come to different conclusions.

As the country with the world’s largest aging population, the impact of China’s aging population on the development of its services industry is already worthy of in-depth study. At the same time, the following patterns of demographic change in China also underline the urgency of the study. First, China is in the early stages of rapid population aging. China’s population aging rate has risen from 7% to 14% in just 20 years, compared with 46 years for Germany, 79 years for the United States and 115 years for France. Second, China’s aging population is huge. By the end of 2021, China had a population of over 267 million people aged 60 and over, and over 201 million people aged 65 and over. The substantial elderly population will have a significant and far-reaching impact on the development of the service industry. Third, China’s aging population is characterised by the phenomenon of "getting old before getting rich". In conclusion, research on China’s aging population and the development of the service industry is more urgent for realistic analysis.

Based on the above analyses, this paper provides theoretical elaborations and empirical tests on population aging and service industry development in China. After a series of robustness tests, this paper found that population aging has a significant "inverted U" effect on the development of the services industry. By region, population aging has a greater impact on economically developed regions than on economically less developed regions. By industry, population aging has a significant impact on the medical and transport industries, while the impact on other service industries is not significant. The threshold effect test showed that population aging has a significant effect on the development of the service industry at a single threshold. Once the degree of population aging exceeds the threshold, the stimulating effect of population aging on the development of the service industry becomes insignificant. The research in this paper provides important insights for dealing with the issue of China’s aging population, optimising the design of service industry development, and building a modernised economic system in the new era.

The marginal contribution of this paper is mainly reflected in the following three aspects: First, in terms of research content, it decomposes from both sides of demand and supply, and proposes the "inverted U" law of the role of population aging in the service industry. This theory provides a new explanation for the contraction pressure of the service industry when population aging accelerates, taking into account the existence of a turning point. Second, in terms of theoretical analysis, previous research analysing the demand and supply effects of population aging has often overlooked the relationship between the elderly and the young, and has not analysed the effects of population aging on the supply and demand of young people. This paper analyses the effects of population aging on the elderly and young population in a more comprehensive and systematic way, so as to clarify the mechanism of population aging on the development of the service industry in a comprehensive and accurate way. Third, in terms of practical application, this paper provides a new explanation for the downward pressure on China’s current economy. It is found that as the degree of population aging increases, the service industry will inevitably be affected. This provides a new way of thinking about the downward pressure on China’s economy, and some insights for deepening the supply-side structural reform of the service industry.

The remainder of the paper is structured as follows: Section 2 provides a review of the relevant literature. Section 3 provides a brief description of the current situation of population aging and the development of the service industry in China. Section 4 presents a theoretical analysis and research hypotheses. Section 5 is a description of the model and data. Section 6 shows the empirical test and results, and Section 7 is the conclusion of the paper.

## Section 2: Literature review

With the intensification of the global trend of population aging, research on the impact of population aging on the development of the service industry has increased. There are two main areas of discussion at present. The first is a study of the economic effects of population aging on the development of services. Some researchers are optimistic about the impact of population aging on the development of the services industry [4, 12]. Using empirical data from 51 countries, including both developing and developed countries, Siliverstovs et al. found that population aging promotes the development of the service industry [13]. As the population ages, the work experience of service workers will be enriched, which will increase the labor productivity of the service industry and promote the rapid development of the service industry [11]. However, some researchers have questioned this. Roger and Wasmer found that high aging reduces labor productivity in services and tends to transfer backward knowledge to future workers [7, 14], which in turn exacerbates the negative effects of aging. As can be seen, research findings on the economic effects of population aging on the development of the services industry are mixed.

In order to clarify the relationship between population aging and the development of the service industry, researchers further focus on the impact path research. The impact path of population aging on the development of the service industry consists of two main aspects. First, the impact of population aging on the demand for services. Estrada et al. selected 31 developing countries in the Asian region and found that population aging contributes to the development of the services industry by positively stimulating domestic demand [4]. In an empirical study based on US household data, Cravion et al. found that population aging can increase household expenditure on services [12]. In another study, Maveov et al. found that aging populations create huge demand for healthcare and eldercare industries [5]. It has been shown that in countries with rapidly aging populations, such as Japan and Finland, aging has been a major driver for the rapid development of the health and leisure services industry [15]. However, some researchers take the opposite view that the increase in the degree of population aging inhibits consumer demand and thus restricts the development of the service industry. For example, Mason et al. found that the aging of the population and the level of consumption of its inhabitants are negatively related [6]. In a study of household consumption in Japan, Wakabayashi found that both the expected and actual consumption of the aging population is much lower than pre-retirement consumption levels [16]. Using data from China, Horioka and Wan found that higher levels of population aging reduce consumption [17].

The second is the impact of population aging on the supply side of service industry development. The impact of population aging on the supply side of services is mainly related to three aspects: the number of labor force, labor productivity and human capital. First, the impact of population aging on the number of labor force, some scholars have found that with the increasing degree of population aging, it will lead to the reduction of age-appropriate labor force [7], which is also empirically proven in China. The decline in the supply of effective labor in society promotes the increase of labor costs in the service industry and hinders the development of labor-intensive industries [8]. In studies of labor productivity, Roger and Wasmer found that labor productivity in the service industry declines with age [7]. Feyrer also found that population aging leads to slower total factor productivity growth and is an important cause of slower output per capita growth [18]. In contrast, Collinson found that the greater work experience of older workers can increase productivity and offset or compensate for the aging effect [19]. In addition, population aging also promotes continuous innovation in labor-substituting technologies as a way of mitigating the adverse effects on the labor supply side [20]. In the study of human capital, Fougère et al. found that population aging can promote the accumulation of human capital, which in turn promotes the development of the services industry [10].

In summary, much attention has been paid to the impact of population aging on the development of the service industry. However, from the above analysis, it is not difficult to identify the following two issues in existing studies. First, research on the development of the service industry is relatively one-sided and cannot comprehensively measure the overall development law of the service industry. Second, the theoretical analysis focuses on the demand and supply analysis of the aging population, while ignoring the primary young issues of consumption and supply in society. Therefore, this paper first constructs a comprehensive development index of the service industry based on the existing scholars’ research. Then in the theoretical analysis, the impact of population aging on the supply and demand of the young population is fully considered. This enables a more comprehensive analysis of the impact of population aging on the development of the services industry.

## Section 3: Facts on aging and service industry characteristics in China

### Trends in the evolution of China’s aging population

Table 1 shows the development of the structure of China’s population aged 65 years and over from 2010 to 2021. As shown in Table 1, from 2010 to 2021, China’s elderly population (aged 65 and over) will increase by 81.62 million, with the aging rate rising by 5.3 percentage points. While the aging rate in India, Brazil and Russia increased by 1.74, 2.67 and 2.91 percentage points respectively over the same period, China’s aging rate is growing rapidly compared to other BRICS countries. From 2016 to 2021, China’s youth dependency ratio will tend to increase following the implementation of China’s fertility policy. From 2010 to 2021, China’s old-age dependency ratio will increase by 8.9 percentage points and the total dependency ratio by 12.1 percentage points. This indicates that the elderly population is growing faster than the working population, which will eventually lead to an increasing dependency burden on China’s working-age population.

**Table 1:** Trends in the evolution of China’s population structure, from 2010 to 2021.

Year	Number of older people (10,000 people)	Aging rate (%)	Child Support Ratio (%)	Old age dependency ratio (%)	Total dependency ratio (%)
2010	11894	8.9	22.3	11.9	34.2
2011	12277	9.1	22.1	12.3	34.4
2012	12777	9.4	22.2	12.7	34.9
2013	13262	9.7	22.2	13.1	35.3
2014	13902	10.1	22.5	13.7	36.2
2015	14524	10.5	22.6	14.3	37.0
2016	15037	10.8	22.9	15.0	37.9
2017	15961	11.4	23.4	15.9	39.3
2018	16724	11.9	23.7	16.8	40.4
2019	17767	12.6	23.8	17.8	41.5
2020	19064	13.5	26.2	19.7	45.9
2021	20056	14.2	25.6	20.8	46.3

Note: The data are from China Statistical Yearbook.

### Projections of the number of aging people in China

As shown in Table 2, China experienced its first rapid population growth in the 1960s, which will lead to a rapid aging of the population for more than 10 years after 2023. There were a total of 134.23 million births between 1962 and 1966, compared with 90.85 million between 1956 and 1960. Under the current pension policy, the number of elderly pensioners in China will increase by 47.7% year-on-year in the next five years. The number of retirees and the rate of aging will rise sharply, putting enormous pressure on China’s economic growth and public services.

**Table 2:** 1962–1971 Births into old age and year of retirement.

Population born 1962–1971 entering old age and year of retirement										
Year of birth	1962	1963	1964	1965	1966	1967	1968	1969	1970	1971
Number of births (million)	2460	2953	2729	2704	2577	2562	2756	2715	2736	2567
Year of retirement	2022	2023	2024	2025	2026	2027	2028	2029	2030	2031
Entering old age	2027	2028	2029	2030	2031	2032	2033	2034	2035	2036

Note: All data are from the China Population and Employment Statistics Yearbook.

### Trends in China’s service industry

**Fig 1:**
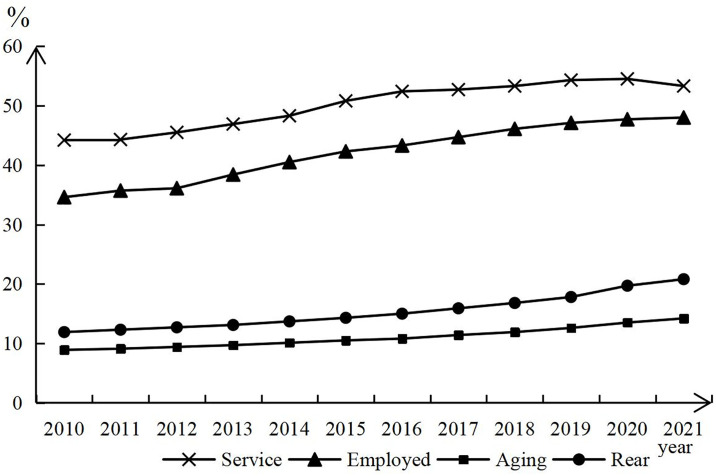
Service industry and aging trends.

Fig 1 shows the evolution of the service industry and population aging in China from 2010 to 2021. As can be seen in Fig 1, the value added of the service industry and the number of people employed in the service industry show an upward trend. From 2010 to 2021, Services value added declines slightly in 2020–2021 due to COVID-19. From 2010 to 2021, the proportion of the service industry workforce increased by 13.4 percentage points. Since 2016, there has been a noticeable acceleration in China’s population aging and the elderly dependency ratio.

## Section 4: Theoretical analysis and research hypotheses

### Demand and supply effects of population aging on the development of the service industry

#### Demand effects of population aging on services development

The impact of population aging on demand for services is reflected both in the level of effective demand for services and in changes in the structure of demand for services. First, with regard to the impact of population aging on the level of effective demand for services. Research on differences in consumption patterns by age shows that older people have a higher demand for services for health and physiological reasons [21], and that this demand for services is fixed and increasing. In addition, due to the time-intensive and non-storable nature of service consumption, older people have more time to consume services. Older people have been found to consume more services than younger people [6, 15]. Therefore, as the population ages, the demand for services in society as a whole will continue to rise. However, as the degree of aging rises excessively, the consumption capacity of the aging population will provide some resistance to the expansion of the scale of demand for services. The study found that the consumption level of retired people does not decline smoothly in retirement. Instead, it declines sharply [22–24]. The aging of the population also has an impact on the consumption of young people and thus on the level of demand for services. On the one hand, the higher aging rate means that the young population has to bear the rising costs of support, which has a direct crowding-out effect on young people’s consumption. On the other hand, the higher cost of support also reduces the current savings rate of young people, which in turn affects the future level of consumption in old age. Kuijs found similar conclusions using Chinese data as a sample [25], and Chamon and Prasad found that the savings rate of China’s urban population has the opposite life-cycle characteristics, i.e. the savings rate of the young population is lower [26].

The second is the impact of population aging on the structure of demand for services. As the population ages, demand for services will shift towards the "silver economy". Demand changes in the aging era should take into account the needs of old age and the whole life cycle. As the population ages, demand for health care and other services will continue to grow and become more rigid, while at the same time stimulating the market potential of the elderly housing, pension insurance, tourism and catering industries. Given the rigidity of the restructuring of demand for services by the aging population. The structure of demand in the service industry will continue to shift towards aging products as the population ages.

In summary, as the degree of aging of the population increases, the demand effect of aging on the services industry will show an increasing and then decreasing trend. In the short term, as the degree of aging rises, the services industry will enter a period of rapid expansion through the dual promotion of consumer demand and investment demand. But in the long run, constrained by the decline in society’s overall consumption capacity, as we enter the phase of excessive aging, the increase in the degree of aging will restrain the further increase in effective demand. Thus, as the degree of aging continues to improve, the demand for services will show a dynamic trend, first increasing and then decreasing, and the structure of demand will change in the direction of aging.

#### Supply effects of population aging on services development

The impact of population aging on the supply side of services is mainly reflected in the supply of labor in services, human capital accumulation and the experience effect of population aging. The first is the impact of population aging on labor supply. Population aging has led to a significant decline in labor market supply and a significant rise in labor costs [27]. Annabi et al. found that labor-intensive enterprises are affected by population aging to increase their production costs [8], which leads to the size of this type of enterprises continue to shrink. The second is the impact of population aging on human capital accumulation. With the increase in life expectancy and the gradual increase in the aging of the population, the expansion of education has become the main way to improve human capital. Using data from OECD countries, Fougère et al. found that population aging can promote the development of the services industry by increasing investment in human capital [10]. However, some researchers have questioned this. Poterba found that population aging has a crowding out effect on investment in education, to the detriment of labor productivity [28]. Lindh using data from OECD countries, also found support for this conclusion [29]. A final look at the impact of population aging on the experience of the service industry. The aging of the population implies a gradual increase in the number of experienced, skilled and highly qualified workers in the older population. Older workers have experience that can offset or compensate for aging factors. Collinson found that knowledge and work experience are embedded in older workers [19], and that rich work experience can help older workers find more effective ways of working [30]. Therefore, in the short term, as the population ages, the negative effects of aging can be offset or compensated for by increasing investment in human capital, promoting the development of labor-substituting technologies and the experience effect of an aging population. However, with the rapid aging of the population, the rapid decline in the number of people in work and the declining rate of human capital accumulation. The negative impact of the supply effect of aging will continue to increase in the long run.

**Fig 2:**
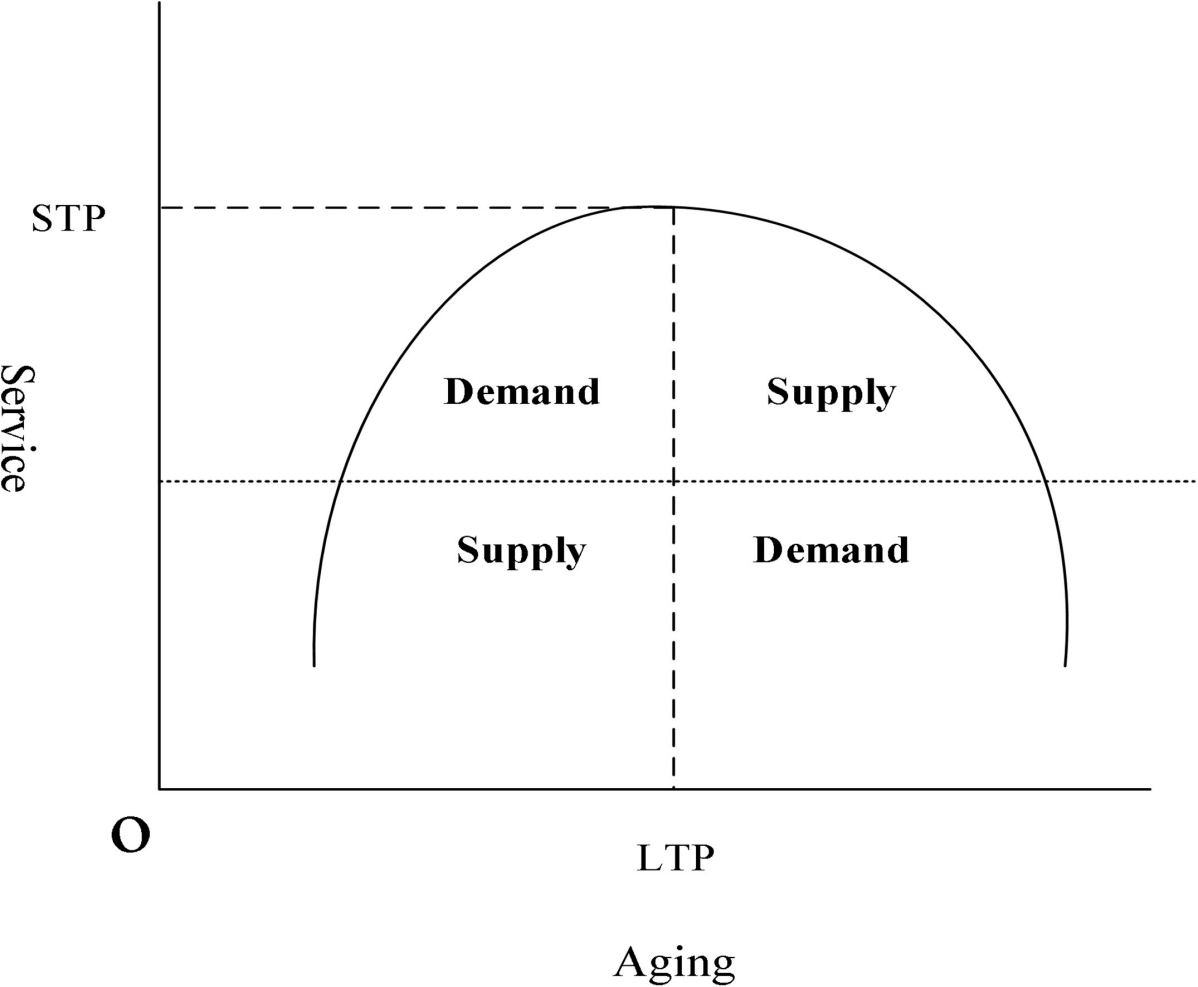
The "inverted U" pattern of the aging population in the service industry.

In summary, the impact of population aging on the development of the services industry will create a supply and demand pull situation. As shown in Fig 2. In the middle and low stages of population aging, the demand effect will be greater than the supply effect, forming the positive promotion effect of population aging on the development of the service industry. If population aging continues to accelerate, it will break the LTP tipping point. The supply effect will gradually be in the ascendant and form a sustained crowded on the demand effect stimulation, and then the development of the service industry breaks through the STP turning point.

In summary, this paper proposes research hypothesis 1: The impact of population aging on the service industry will show a dynamic change of "promotion" followed by "inhibition".

### Heterogeneity analysis

The overall impact of population aging on the services industry is "inverted U" shaped, but there are differences in performance in the face of regional imbalances and industry heterogeneity. Analysis of cross-country data reveals a "two-wave" evolution of the services industry, depending on the level of economic development [31]. Regions with a high level of economic development are characterised by higher purchasing power and greater consumer demand, which will "internalise" the new demand created by aging. The "good digestion" of consumer demand will influence the direction of industrial development, which will further influence the industrial structure through the transmission effect. In economically developed areas, the level of per capita income and consumer demand is high, and the level of urbanisation is usually higher, which is more conducive to the development of modern service agglomerations. In economically underdeveloped areas, the level of per capita income is relatively low, and consumption is mostly focused on daily necessities, with little consumption demand for the pleasure-oriented services. In less developed areas, per capita income levels are low, urbanisation levels are low, and the service industry is concentrated in traditional services, making it difficult to create effective demand for modern services. Economically developed regions will also attract a greater concentration of skilled labor due to their good infrastructure and well-developed market systems, thus offsetting to some extent the negative impact of the decline in the labor force due to aging.

In summary, this paper proposes research hypothesis 2: compared to economically underdeveloped areas, the aging of the population in economically developed areas has a more pronounced effect on promoting the development of the service industry.

The aging of the population has skewed consumer demand and also determines the heterogeneity of the impact on different industries. The aging population has its unique consumption demand and labor supply capacity, especially consumption is more inclined to the demand of consumer service industry, such as medical and health industry, tourism and so on. The economic risk tolerance of the aging population will gradually decrease due to the decline in economic income. This means that there is less product demand for the financial, real estate and other investment characteristics of the industry. The aging population on the supply side of the impact of significant industry heterogeneity. First, as the population ages, the supply of labor in manual industries such as real estate will decline more sharply. Second, the re-employment of the aging population is more inclined towards knowledge-based services, relying on manual labor and other industries is difficult to re-employ. In summary, population aging will have a heterogeneous impact on different service industries.

In summary, this paper proposes research hypothesis 3: Population aging has a heterogeneous impact on the development of different service industries.

## Section 5: Model construction and data description

### Model construction

In this paper, we use the fixed effects model to examine the impact of population aging on the multidimensional factors of the service industry. Given the possible non-linear relationship between population aging and the development of services, we introduce the quadratic aging term into the model. At the same time, in order to further test whether population aging will have a dynamic impact on the development of the service industry, first "promoting" and then "inhibiting", this paper refers to the relevant studies by Hansen to construct a panel threshold regression model [32]. The specific regression model is as follows.

$$lnservice_{it}=\alpha_{0}+\alpha_{1}\times aging_{it}+\alpha_{2}\times aging_{it}^{2}+\beta \times Controls_{it}+u_{it}$$ (1)

$$\begin{array}{c} lnservice_{it}=\alpha_{0}+\alpha_{1}\times aging_{it}\times I(aging_{it}\leq T)+\alpha_{2}\times aging_{it}^{2}\times I(aging_{it}\leq T)+\alpha_{3}\times aging_{it}\times \\ I(aging_{it}>T)+\alpha_{4}\times aging_{it}^{2}\times I(aging_{it}>T)+\beta \times Controls_{it}+u_{it} \end{array}$$ (2)

where, *i* and *t* denote region and time, respectively; *lnservice*_*it*_ is the Services Development Index, which means the service industry expansiveness index. Next, *aging*_*it*_ is the explanatory variable, which indicates the degree of population aging, specifically meaning the proportion of the population aged 65 years or over in the total population in i province in year *t*; *I*(⋅) is the indicator function of the threshold estimation model, and *T* is the threshold value. *Controls*_*it*_ is the control variable, and *μ*_*it*_ indicates the random disturbance term.

Dependent variable: Services Development Index (*lnservice*_*it*_). This paper uses the entropy value method to creatively construct a service industry expansiveness indicator, which in turn is borrowed to measure the overall development of the service industry. The indicator consists of three specific indicators, namely the number of service industry companies, the number of people employed in the service industry, and the value added to the service industry.

Independent variable: Degree of population aging (*aging*_*it*_). The degree of aging of the population is measured by the proportion of people aged 65 and over in the total population. China’s demographic structure will lead to an acceleration of the degree of aging. Since around 2000, China has entered an aging society, the country has been ranked among the top countries of the same level of development in terms of the rate of aging, reaching 14.9% of the population aged 65 and over in 2022. Now, China is entering an era of a deeply aging society. China will move from an aging society to a deeply aging society in just over 20 years, significantly shorter than the world average of 35 years. In the future, China’s aging rate will accelerate further and be accompanied by a retirement peak, thereby exacerbating the social problems associated with aging. This paper also uses the elderly dependency ratio (*aging*_*r*_) as an explanatory variable to check the robustness of the results.

Control variables: 1. Social Security Level (*peo*). The degree of social security is expressed as the ratio of the number of pensioners to the resident population at the end of the year (unit: %). The level of social security is an important factor influencing the reemployment intentions and consumption of the older population [33]. The increase in the level of social security can promote the improvement of the actual consumption capacity of the older population and stimulate the development of the service industry on the demand side. 2. Urbanization Rate (*urban*). The consumption of services is time-intensive and non-storable, among other characteristics, so the development of the service industry requires the clustering of product consumers and producers within a certain range. The development of urbanisation provides favourable conditions for the clustering of the two [34], so the development of urbanisation will have a certain impact on the development of the service industry (unit: %). 3. Human Capital Level (*eduyear*). The level of human capital is measured using years of schooling. The core input factor in the production process, labor, which can greatly affect the development of industry, advanced human capital is the source of power that allows the development of modern industries. McMillan found that the development of services in developing countries depends to some extent on the level of human capital [35]. 4. Total Population (*population*). The total population measure at the end of the regional year is used here (unit: billion persons). 5. Child Dependency Ratio (*cdr*). As the child dependency ratio affects the demand and supply of services by households, this paper adds the child dependency ratio as a control variable (unit: %). 6. Regional GDP per Capita (*pgdp*). Regional GDP per capita is related to the consumption level and demand size of regional residents, which in turn will affect the demand size of the service industry (unit: million yuan). 7. Labor Force Population (e*mployment*). Regional private industry and self-employment is used here as a measure. The movement of the regional labor force between different industries may also affect the development of the service industry, so the regional labor force population is controlled for in this paper (unit: billion persons). Table 3 shows the descriptive statistics of the main variables.

**Table 3:** Descriptive statistics.

variable	Observations	mean	std	min	max
lnservice	480	6.300	0.970	3.752	8.772
lncorporate	480	7.767	0.971	5.225	10.228
lnlabor	480	2.202	0.631	0.649	3.652
lnservicegdp	480	3.877	1.000	1.226	6.540
aging	480	10.373	2.506	5.473	18.805
peo	480	0.492	0.217	0.524	0.922
urban	480	56.000	13.600	27.500	89.600
eduyear	480	8.963	1.005	6.594	12.782
population	480	0.453	0.278	0.054	1.268
cdr	480	23.297	6.548	9.640	42.220
pgdp	480	4.162	3.000	0.610	18.398
employment	480	0.099	0.107	1.005	0.707

### Data sources and processing

This paper selects the panel data of 30 provinces in China from 2006 to 2021 for empirical analysis (excluding the Tibet Autonomous Region). The relevant data are taken from China Statistical Yearbook, China Tertiary Industry Statistical Yearbook and China Population and Employment Statistical Yearbook; the relevant economic variables are deflated using 2006 as the base period to eliminate the effect of price fluctuations. In addition, the relevant variables are treated as logarithmic in the model to reduce the effect of heteroskedasticity.

## Section 6: Empirical tests

### "Inverted U" type test and analysis

First, the multidimensional impact of population aging on the service industry is empirically analysed using Model 1. The coefficient of the primary term in column 1 of Table 4 is significantly positive and the coefficient of the quadratic term is significantly negative, indicating that population aging plays a dynamic change characteristic of promoting and then inhibiting the development of the service industry. Columns 2 to 4 show the results of the tests on the value added of the service industry, the number of people employed in the service industry and the number of companies in the service industry. The results show that the primary coefficients are significantly positive, and the secondary coefficients are significantly negative. The multi-dimensional test results are robust, and the "inverted U" type relationship is significant. This finding indicates the existence of a significant multidimensional effect of aging on the service industry, with all indicators first promoting the development of the service industry, and then inhibiting the further development of the service industry when aging reaches a certain threshold. Prior to the regression, the paper conducted a Hausman test, which supports the superiority of using a fixed effects model for estimation.

**Table 4:** Basic regression results.

	(1)	(2)	(3)	(4)
	lnservice	lnservicegdp	lnlabor	lncorporate
aging	0.167***	0.091**	0.090***	0.174***
	(4.16)	(2.08)	(2.84)	(4.12)
aging^2^	-0.006***	-0.004*	-0.004***	-0.007***
	(-3.82)	(-1.91)	(-3.32)	(-3.79)
peo	0.081	-0.030	-0.039	0.088
	(0.64)	(-0.22)	(-0.32)	(0.65)
urban	5.143***	3.450***	0.977*	5.265***
	(9.02)	(5.80)	(1.92)	(8.50)
eduyear	0.064	0.034	0.064**	0.066
	(1.29)	(0.88)	(2.45)	(1.25)
pgdp	0.076***	0.131***	0.036***	0.072***
	(4.61)	(11.64)	(3.53)	(3.88)
population	0.000***	-0.000***	0.000**	0.000***
	(6.73)	(-3.25)	(2.53)	(6.90)
cdr	0.006	-0.002	-0.002	0.006
	(1.19)	(-0.33)	(-0.39)	(1.21)
employment	0.000	0.000***	-0.000	-0.000
	(0.03)	(6.31)	(-0.32)	(-0.32)
constant	0.109	1.686***	0.134	1.285*
	(0.17)	(3.78)	(0.27)	(1.89)
Time fixed effects	Yes	Yes	Yes	Yes
Individual fixed effects	Yes	Yes	Yes	Yes
*N*	480	480	480	480
R^2^	0.952	0.951	0.751	0.946

Note: t-values are in brackets,Control variables are the same below.

***p<0.01.

**p<0.05.

*p<0.1.

The stage heterogeneity of aging’s impact on services is significant. The negative coefficients of the quadratic terms indicate that the marginal contribution of population aging to the development of the service industry is gradually diminishing and may even have an inverse restraining effect. Meanwhile, population aging has a convergent effect on three factors within the service industry: the number of service enterprises, the gross value of services and the number of persons employed in services.

In the primary stage of aging, the gross service industry product, the number of service industry employees and the number of service industry firms are in an expansionary phase as aging deepens. During the primary stage of aging, the increasing demand for health care, recreation, leisure and tourism services from an increasingly elderly population leads to an expansion in the scale of production in the service industry. This industry then absorbs a large amount of new labor, due to the expansion in production. When labor costs rise as a result of the reduction of the labor force, enterprises are forced to carry out technological innovation. In addition, part of the labor force released by technological substitution flows to the service industry, which has high employment elasticity. This results in a significant increase in the number of people employed in the service industry. As the population ages, a number of service industries closely related to aging will gradually emerge in the early to medium term and enter a period of rapid development..

If the aging of the population continues to deepen, the restraining effect of aging on the service industry will become the main effect, hindering the development of the service industry. The labor force participation rate of the over-65s population is relatively low, and the consumption of this segment of the population relies on old-age pensions, intergenerational transfers, and the savings of their younger years. As the population ages, limited economic resources will lead to a decline in real consumption capacity. In addition, the uncontrolled aging of the population will lead to a shift in China’s demographic structure from an olive to an inverted triangle, with a declining workforce in the service industry. At present, the aging market tends to be saturated, competition is intensifying, and the market environment facing companies is deteriorating. The aging of the population has changed the development of service industry enterprises from initial promotion to inhibition, forcing some enterprises to withdraw from the market in the face of fierce market competition. In summary, the aging of the population has a multidimensional impact on the service industry and the development of stage heterogeneity, which generally shows the order of promotion and then inhibition.

### Robustness test

#### Substitution of explanatory variables

This article uses the share of the population aged 65 and over in the total population in the benchmark regression to measure the aging rate of the population. In addition to this indicator, the old-age dependency ratio is also a commonly used indicator to measure the aging rate of the population. To further increase the robustness of the conclusions of the article, this paper uses the old-age dependency ratio to measure the aging rate for robustness testing. The results, as shown in Table 5 (1), show that the "inverted U" effect of population aging on the development of the services industry is still significant, which to some extent proves the robustness of the conclusions of this paper.

**Table 5:** Robustness test.

	(1)	(2)	(3)	(4)	(5)	(6)
	lnservice	lnservice	lnservice	lnservice	lnservice	lnservice
aging		0.068*	0.157***	0.390***	0.172***	0.389**
		(1.73)	(3.99)	(10.17)	(6.73)	(2.44)
aging^2^		-0.004**	-0.006***	-0.015***	-0.006***	-0.015**
		(-2.41)	(-3.41)	(-9.40)	0.172***	0.389**
aging_r_	0.100***					
	(4.84)					
aging_r_^2^	-0.003***					
	(-4.61)					
constant	0.289	2.796***	0.067	1.384***	0.165	-0.609
	(0.48)	(3.77)	(0.11)	(3.86)	(0.40)	(-0.92)
Control variables	Yes	Yes	Yes	Yes	Yes	Yes
Time fixed effects	Yes	Yes	Yes	Yes	Yes	Yes
Individual fixed effects	Yes	Yes	Yes	Yes	Yes	Yes
*N*	480	480	450	480	464	450
R^2^	0.952	0.962	0.939	0.910	0.956	0.931

#### Interaction fixed effects

The impact of population aging on the development of the services industry may also be influenced by other unobservable factors. For example, certain national or local policies aimed at promoting demographic change or developing services. These may bias the estimated results. To further increase the robustness of the conclusions, this paper adds the interaction terms of region fixed effects and year fixed effects to further control for the impact of regional factors on the development of the services industry. The regression results are presented in Table 5 (2) and there is no significant change from the benchmark regression results, further demonstrating the robustness of the conclusions of this paper.

#### Lag effect test

Consideration of the possible lagged effects of population aging on the development of services. In order to test the dynamic effect of population aging on the development of the service industry, this paper simultaneously conducts a lagged one-period regression of the dependent variable and the control variable. The results are shown in Table 5 (3). The lagged one-period regression results are consistent with the benchmark regression results, further validating the robustness of the paper’s conclusions.

#### Serial correlation and heteroskedasticity test

Considering that the sample data in this paper may have serial correlation and heteroskedasticity problems, which may lead to the bias of the benchmark regression results in this paper. Therefore, the serial correlation test and the heteroskedasticity test are carried out in this paper. The test results show that there is no serial correlation in this paper. White’s test shows that there is heteroskedasticity in this paper. To further eliminate the effect of heteroskedasticity on the benchmark regression results, this paper also uses the WLS method to estimate the test. The results are shown in Table 5 (4). After eliminating the effect of heteroskedasticity, the "inverted U" shape of population aging on the development of the service industry is still significant, which proves the robustness of the conclusions of this paper.

#### Instrumental variables test

Although this paper has better controlled for omitted variables and endogeneity, there may still be a bi-directional causality between population aging and the development of the services industry. Therefore, this paper tries to eliminate the endogeneity problem by using the instrumental variables method. This paper selects the following two instrumental variables for regression analysis. First, the fertility rate from China’s fourth census data is selected as an instrumental variable for population aging. On the one hand, the historical fertility rate is a better predictor of the degree of population aging [36], and the use of this instrumental variable satisfies the correlation assumption. On the other hand, it is generally accepted that historical fertility rates are unlikely to have a direct impact on the development of the current service industry, and even if they do, it is only through the demographic channel. Therefore, it also satisfies the exclusivity assumption. It is a more qualified instrumental variable. The use of historical fertility as a demographic instrumental variable is also more common in the literature [37]. To further validate the endogeneity issue in this paper, the lagged one-period variable of the population aging rate is selected to be tested again as an instrumental variable for population aging. The results are shown in columns 5 and 6 of Table 5. After adding the instrumental variables to the regression model, the results are not significantly different from the original regression results. The instrumental variables in this paper passed the weak instrumental variable test and the unidentifiable test, which proves the validity of the instrumental variables in this paper.

### Threshold effect test

Before using the threshold effect model, the first step is to analyse the existence of the threshold effect model. The results are shown in Table 6. This paper conducts the threshold effect analysis of the population aging rate, and after the self-help method of repeated sampling for 500 times, the results show that the population aging rate significantly passes the single-threshold test and fails to pass the double-threshold test, so this paper analyses the use of a single-threshold model. From the estimation results of the threshold effect in Table 7, it can be seen that when the population aging rate exceeds the threshold, the stimulating effect of population aging on the service industry is no longer significant.

**Table 6:** Existence test for threshold effect.

	Thresholds	F value	P value	threshold value	confidence interval
lnservice	Single Threshold	37.48**	0.023	15.162	(14.628,15.467)
	double threshold	13.93	0.363		
lnservicegdp	Single Threshold	12.90**	0.030	12.902	(12.426,12.986)
	double threshold	9.13	0.407		
lnlabor	Single Threshold	42.86***	0.001	14.587	(14.279,14.976)
	double threshold	11.25	0.257		
lncorporate	Single Threshold	15.16**	0.017	14.634	(13.975,15.031)
	double threshold	13.08	33.917		

**Table 7:** Results of threshold effect estimation.

	lnservice	lnservicegdp	lnlabor	lncorporate
Aging I(Th≤T)	0.113*(2.03)	0.113*(1.72)	0.119**(2.65)	0.341***(4.21)
Aging I(Th>T)	1.193(1.33)	-0.117(-0.29)	0.056(0.52)	0.093(0.28)
Control variables	Yes	Yes	Yes	Yes
Time fixed effects	Yes	Yes	Yes	Yes
Individual fixed effects	Yes	Yes	Yes	Yes
*N*	456	398	446	433
R^2^	0.951	0.947	0.773	0.888

Note: N and R^2^ are reported here when the population aging rate is below the threshold.

### Simulation of the effects of the coming "retirement peak"

**Fig 3:**
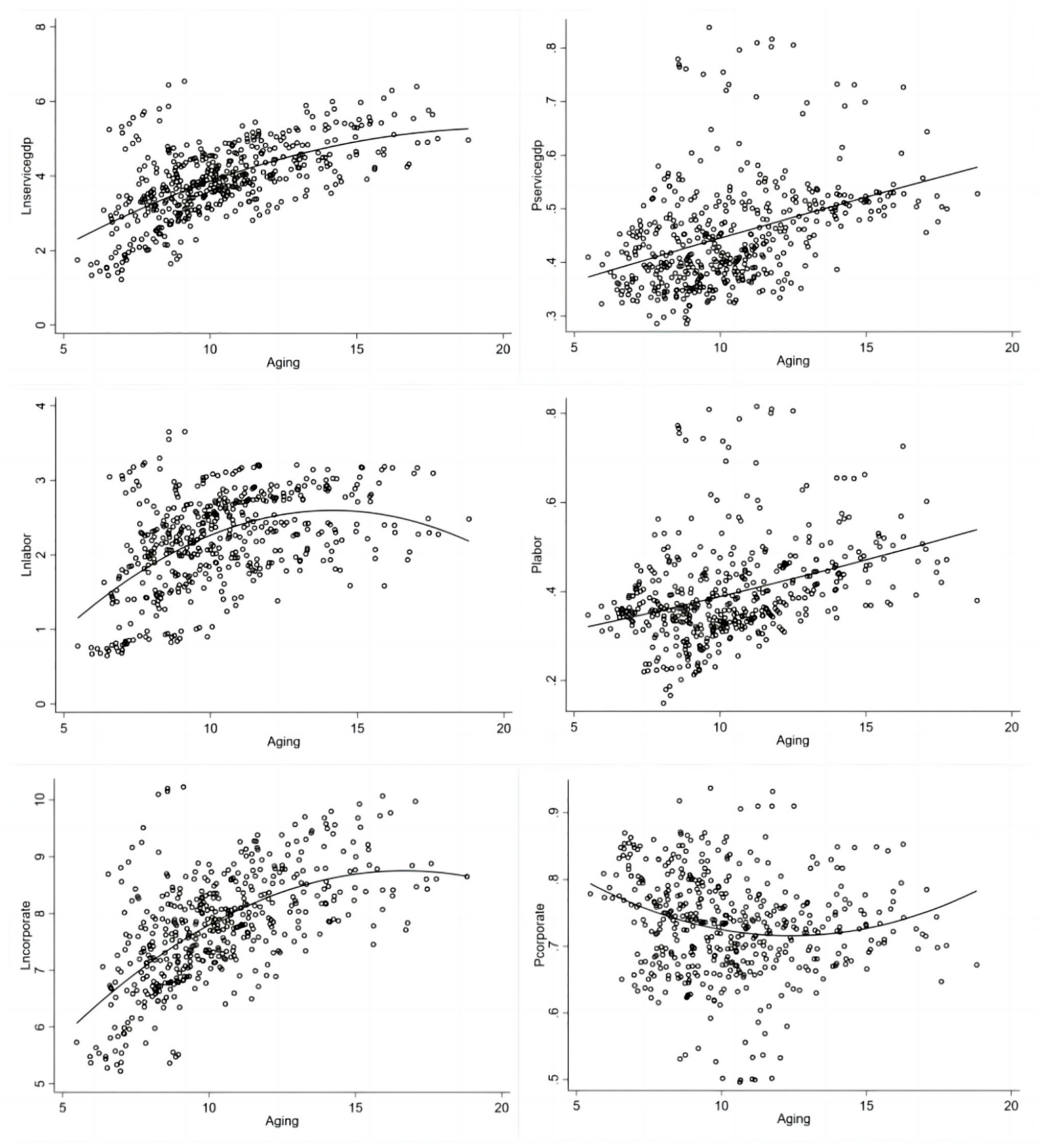
Plot of linear fit of aging and services.

China’s population aging will experience an accelerated phase from 2020 to 2030. On the basis of China’s aging rate in the past decade, China will reach 15.81% in 2025, already indicating a deeply aging society. In reality, the aging level will be higher than this value. The next decade will be a serious period of accelerated aging, which will exacerbate the "retirement peak". Therefore, to further simulate the future "retirement peak" scenario, this paper constructs a simulation of the degree of aging and the above three indicators, as shown in Fig 3.

The "double U" shape effect of aging on the three factors is significant: an "inverted U" shape in terms of quantity, and a "positive U" shape in terms of proportion. The fitted graph shows that the majority of provinces in China are still at the left end of the "STP" point. In those provinces, aging still has a catalytic effect on the development of the service industry. By factor, the impact of aging on the development of the service industry is first seen at the employment level. This suggests that the service industry is already experiencing a labor shortage as aging continues to increase. However, at present, the impeding effect of aging on the gross value of the service industry and the expansion of the number of service industry companies is not yet apparent. The next 10 years will coincide with the retirement peak. During this period, China’s population of over-65s is expected to increase by 243 million, and the proportion of elderly people will increase by 9%. Faced with the impact of the retirement peak, the number of companies in the service industry will rapidly cross the inflection point and turn downward. Meanwhile, the number of people employed in the service industry will be in a shrinking situation, which will have an all-round effect on the expansion of the service industry.

The results of the linear fit between aging and the share of the three factors mentioned above are quite different, showing a positive ’U’ shaped relationship. Given the current stage of aging, a deepening of aging will lead to a structural shift to a service-oriented economy from the three factors of employment, business and gross product in all directions. As the size of the aging population increases, the overall resources of society will continue to shift towards the service industry. The first direct manifestation of this is the increasing shift of labor to the service industry. This result is consistent with both the reality in China and the expectations of Clark’s theorem. Secondly, the shift in the consumption structure of the aging population will indirectly stimulate an increase in the supply of service providers and the volume of supply. In other words, there will be an increase in the proportion of service industry companies and the proportion of service industry output in the gross national product. The onset of the retirement peak will lead to a more rapid change in the consumption structure and will undoubtedly accelerate the process of transferring social resources to the service industry. At the same time, as the service industry becomes the dominant industry in China’s economy, given the service industry inefficiency (and the difficulties already highlighted in terms of improving its efficiency), China’s economy will have difficulty maintaining stable growth in the future.

From top to bottom, the linear fits are shown for the three indicators of GDP of the service industry, the number of service industry workers and the number of service industry firms respectively, with the linear fits for the absolute quantities of the three indicators on the left and the linear fits for the relative quantities of the three indicators on the right.

### Analysis of heterogeneity effects

The degree of population aging has a significant regional and industry heterogeneity on the development of the service industry. This paper now examines the heterogeneity from two perspectives: regional heterogeneity and industry heterogeneity.

Firstly, the 30 provinces of China are classified into economically developed regions and economically less-developed regions, according to the regional GDP per capita. Specifically, when the regional GDP per capita exceeds three-quarters of the quartile, the region is considered economically developed, and vice versa. We then empirically analysed the regional heterogeneity of the impact of population aging on the development of the service industry, and the results are shown in Table 8. The "inverted U" type effect of population aging on the development of the service industry is significant. Compared to economically less developed regions, the favourable effect of population aging on the development of the services industry is stronger in economically developed regions. The possible reason for this is that the effective demand in economically underdeveloped regions is difficult to support the development of regional service industries, and there is a phenomenon of outflow of consumption in medical treatment, recreation and health care, and other types of consumption. The labor capital in economically underdeveloped regions is relatively low, and it is difficult to form a reserve of talents for the development of modernised service industries, and the improvement of labor efficiency is even slower. Specifically, the level of the regional economy is closely linked to the degree of development of the service industry and is also influenced by both supply and demand. Firstly, looking at the impact of regional demand on the service industry from a demand perspective, one can see that economically developed regions have higher income levels and better social security systems. In addition, the income effect drives a stronger willingness to consume among the aging population. In contrast, in economically less-developed regions, income levels are lower, and savings levels are lower. Most of the elderly population’s income is used to support survival needs and to set aside certain funds to offset medical expenses. As a result, the effective demand for other services, especially enjoyment services, is basically zero. Secondly, from the supply side, one can observe the role of regional supply levels in driving the service industry. From the perspective of labor supply, economically developed regions are rich in high-quality labor resources. Also, labor productivity declines slowly, and these regions have a stronger ability to learn and accept digital technology, which is conducive to the structural upgrading and efficiency optimisation of the service industry. In addition, the comprehensive attractiveness of economically developed regions to labor ensures a constant supply of high-quality labor. From the perspective of infrastructure development, economically developed regions have better infrastructure and higher levels of urbanization, both of which are conducive to the agglomeration development of the service industry, helping to realize the scale effect of the service industry and reduce costs. The service industry in economically less-developed regions is mostly at the traditional stage, which means the industry is characterized by low efficiency and low value-added. Also, in the poorer regions, there is a serious problem of population exodus. The analysis of the regional heterogeneity of the impact of aging on the service industry shows that significant differences exist between regions. In addition, the economic gap between regions may be further widened through the development of the service industry.

**Table 8:** Regional heterogeneity in the impact of aging on the service industry.

	(1)	(2)
	lnservice	lnservice
aging	0.156**	0.204***
	(2.38)	(5.60)
aging^2^	-0.008***	-0.006*
	(-4.43)	(-1.98)
constant	0.896	-1.959
	(1.05)	(-1.56)
Control variables	Yes	Yes
Time fixed effects	Yes	Yes
Individual fixed effects	Yes	Yes
*N*	359	121
R^2^	0.940	0.898

Note: t-values are in brackets

***p<0.01.

**p<0.05.

*p<0.1.

### Industry heterogeneity

This paper fully takes into account the group characteristics of the aging population, i.e. the elderly population has different consumption tendencies and sensitivities for different service industries. Thus, the aging of the population will have obvious heterogeneous impacts on different service industries. Due to the difficulty in obtaining data, it is difficult to construct the development indicators of the services sub-industry in this paper, so we use the value added of the services sub-industry to measure the development of the services industry side-by-side. Due to the different consumption habits of the aging population in different industries. For example, demand in the medical industry increases with age and is rigid, while demand in riskier industries decreases with age. On the supply side, there will also be an influx of labor into industries with higher demand. This means that some industries will show positive demand and supply growth at the same time. This paper therefore assumes that the impact of population aging on the growth of the various service industries will be linear. This paper uses Model (3) to check that the variables are consistent with Model (1).


$$lnservice_{it}=\alpha_{0}+\alpha_{1}\times aging_{it}+\beta \times Controls_{it}+u_{it}$$ (3)

As shown in Table 9, the aging of the population has a significant positive impact on the medical and transport industries. Most empirical studies have also shown that an aging population can effectively promote the rapid development of the domestic medical industry [5]. And the effect of population aging on the financial, real estate and catering industries is not significant. According to life-cycle theory, people will rationally accumulate their wealth and maximise their utility over their lifetimes. The "security of the heart" in old age is at a lower level, with a strong aversion to risky investment behaviour, which indirectly affects the development of investment-oriented service industries. China’s current elderly population is still in the state of "getting old before getting rich". Social security can only meet the basic pension needs of the population and cannot significantly improve the disposable income of the population. As a result, the impact of aging on the development of the financial, real estate and other service industries will be limited.

**Table 9:** The impact of aging on the heterogeneity of the service industry.

	(1)	(2)	(3)	(4)	(5)	(6)	(7)
	Finance	Retail	Catering	Transportation	Real Estate	Medical	Other
aging	0.026	0.073*	0.016**	0.066**	0.008	0.065***	0.178***
	(1.56)	(1.92)	(2.72)	(2.48)	(0.78)	(2.87)	(3.81)
constant	1.467**	2.181	0.281	1.669**	0.988**	0.238	2.003
	(2.45)	(1.34)	(1.59)	(2.10)	(2.50)	(0.25)	(1.15)
Control variables	Yes	Yes	Yes	Yes	Yes	Yes	Yes
Time fixed effects	Yes	Yes	Yes	Yes	Yes	Yes	Yes
Individual fixed effects	Yes	Yes	Yes	Yes	Yes	Yes	Yes
N	416	416	416	416	416	416	416
R^2^	0.773	0.667	0.655	0.761	0.608	0.723	0.751

Note: t-values are in brackets

***p<0.01.

**p<0.05.

*p<0.1.

In addition, in order to avoid the effect of COVID-19, this paper uses sample data from 2006 to 2019 for the regression analysis. The study found that the aging of the population has only a non-significant impact on the financial and real estate industries, both of which are in a position to promote the development of other service industries. Following the impact of COVID-19, China’s demand for services has been affected to some extent, but the ’inverted U’ pattern of the impact of population aging on services still exists.

## Section 7: Conclusion and policy recommendations

This paper examines the impact of population aging on the development of service industries in the context of rapid population aging. Theoretically, this study infers that population aging on the whole may have an "inverted U" shape effect on the development of the service industry. This effect first promotes and then inhibits the development of the service industry. This study also takes into account the regional disparities in economic development and the differences in the sensitivity of the service Industries of the aging population, which may have heterogeneous effects on the impact of population aging on the service industry. Therefore, this paper, using a fixed effects model, conducts a multi-dimensional analysis of the value added, employment, number of enterprises and service industry development index, based on provincial panel data from 2006 to 2021. The study found that: (1) The aging of the population in the service industry has an "inverted U" type relationship of promotion and then inhibition, is now close to the tipping point and faces the challenge of "retirement peak". (2) Compared to economically less developed regions, the favourable effect of population aging on the development of services is more pronounced in economically developed regions. (3) Population aging can significantly promote the development of transport, medical and other immediate needs of the service industry, the financial, real estate and other investment industries have no significant impact. (4) The threshold effect test shows that when the population aging rate exceeds the threshold, the promoting effect of population aging on the service industry is no longer significant.

Based on the above findings, there are the following policy implications: (1) Along with the phased adjustment of the population structure, China has taken a proactive approach to seizing the economic growth opportunities embedded in aging. China is catering to the market demand of the elderly population, and adjusting the layout of the service industry, gradually tilting towards medical care, recreation and leisure tourism. Aging service products are also being provided. Nevertheless, the elderly service system should be strengthened and improved, and the government should be aware of the impact of rapid population aging on medical and other demand Industries. The elderly service system should be improved by building a long-term mechanism for elderly services and mitigating the risks of elderly care. A three-dimensional family-enterprise-society maternity support policy should be promoted, including three major policy tools: childcare services, child allowances and paid parental leave. This would ease the burden of childbirth and old age on the population of childbearing age. (2) The state should adopt a forward-looking response strategy to mitigate the impact of the decline in absolute labor force numbers and labor productivity caused by the aging population. Steps should be taken to accelerate the improvement of the delayed retirement policy system, strengthen the regulation of the re-employment market for the retired population, and increase the willingness of the retired labor force to re-employ. Full play should be given to the role of a reservoir for the aging labor force. The government should also accelerate the organic combination of human capital and high technology, improve labor productivity, and accelerate the transformation and upgrading of the manufacturing industry and the structural optimization of the service industry. (3) Promote technological progress and increase investment in human capital. When the degree of population aging exceeds the threshold, the negative impact of population aging on the development of the service industry seems inevitable. China also faces a rapidly aging population. Therefore, the local government should intervene in the rate of technological progress and strengthen investment in human capital to prevent the strong impact on the service industry caused by the rate of technological progress lagging behind that of population aging.
